# Breaking Tolerance in Transgenic Mice Expressing the Human TSH Receptor A-Subunit: Thyroiditis, Epitope Spreading and Adjuvant as a ‘Double Edged Sword’

**DOI:** 10.1371/journal.pone.0043517

**Published:** 2012-09-07

**Authors:** Sandra M. McLachlan, Holly A. Aliesky, Chun-Rong Chen, Gao Chong, Basil Rapoport

**Affiliations:** Thyroid Autoimmune Disease Unit, Cedars-Sinai Research Institute and UCLA School of Medicine, Los Angeles, California, United States of America; Cardiff University, United Kingdom

## Abstract

Transgenic mice with the *human* thyrotropin-receptor (TSHR) A-subunit targeted to the thyroid are tolerant of the transgene. In transgenics that express low A-subunit levels (Lo-expressors), regulatory T cell (Treg) depletion using anti-CD25 before immunization with adenovirus encoding the A-subunit (A-sub-Ad) breaks tolerance, inducing extensive thyroid lymphocytic infiltration, thyroid damage and antibody spreading to other thyroid proteins. In contrast, no thyroiditis develops in Hi-expressor transgenics or wild-type mice. Our present goal was to determine if thyroiditis could be induced in Hi-expressor transgenics using a more potent immunization protocol: Treg depletion, priming with Complete Freund's Adjuvant (CFA) + A-subunit protein and further Treg depletions before two boosts with A-sub-Ad. As controls, anti-CD25 treated Hi- and Lo-expressors and wild-type mice were primed with CFA+ mouse thyroglobulin (Tg) or CFA alone before A-sub-Ad boosting. Thyroiditis developed after CFA+A-subunit protein or Tg and A-sub-Ad boosting in Lo-expressor transgenics but Hi- expressors (and wild-type mice) were resistant to thyroiditis induction. Importantly, in Lo-expressors, thyroiditis was associated with the development of antibodies to the *mouse* TSHR downstream of the A-subunit. Unexpectedly, we observed that the effect of bacterial products on the immune system is a “double-edged sword”. On the one hand, priming with CFA (mycobacteria emulsified in oil) plus A-subunit protein broke tolerance to the A-subunit in Hi-expressor transgenics leading to high TSHR antibody levels. On the other hand, prior treatment with CFA in the absence of A-subunit protein inhibited responses to subsequent immunization with A-sub-Ad. Consequently, adjuvant activity arising *in vivo* after bacterial infections combined with a protein autoantigen can break self-tolerance but in the absence of the autoantigen, adjuvant activity can inhibit the induction of immunity to autoantigens (like the TSHR) displaying strong self-tolerance.

## Introduction

Transgenic mice with the A-subunit of the human thyrotropin receptor (TSHR) targeted to the thyroid gland exhibit tolerance to the transgene. Thus, unlike wild type littermates, the transgenics do not respond to immunization with a low dose of adenovirus encoding the autoantigen, the human TSHR A-subunit [1]. Two transgenic lines express different amounts of TSHR A-subunit in the thyroid gland and the thymus [2], [3]. Because of different expression levels in the thymus, these mouse lines have different levels of self-tolerance to the human A-subunit. In low-expressor A-subunit transgenics (Lo-expressors), tolerance is readily broken using high doses of A-subunit adenovirus in terms of antibody generation. In contrast, transgenic mice that express high levels of the transgene (Hi-expressors) generate little or no TSHR antibody in response to high dose A-subunit adenovirus immunization, even if pre-treated with anti-CD25 to deplete regulatory T cells (Treg)[2], [3]. Much higher TSHR antibody levels can be induced in Hi-expressor transgenics using a more aggressive approach, namely immunization with A-subunit protein emulsified in complete Freund's adjuvant (CFA) followed by A-subunit protein in incomplete Freund's adjuvant [1]. However, TSHR antibodies induced using adjuvant are non-stimulatory and do not induce hyperthyroidism.

Previously, we observed that Lo-expressor transgenics depleted of CD25 positive cells before immunization with TSHR adenovirus (A-subunit or holoreceptor) developed massive thyroid lymphocytic infiltration and thyroid damage associated with hypothyroidism and autoantibody spreading to the other two major thyroid autoantigens, thyroglobulin (Tg) and thyroid peroxidase (TPO)[2], [4]. This dramatic outcome was not observed in wild-type littermates depleted of CD25 positive cells and immunized in the same way. Analysis of T cell and antibody responses revealed that the basis for thyroid lymphocytic infiltration in Lo-expressor transgenics (but not in wild-type littermates) was the presence in the target organ of the immunogen, namely the *human* A-subunit [4].

Very high levels of human A-subunit protein are present in thyroid tissue of the Hi-expressor transgenics [3]. Consequently, it seemed possible that thyroiditis would be induced in the Hi-expressor transgenics, assuming that tolerance could be broken at the T cell level. Both Lo- and Hi-expressor transgenic mice are on the BALB/c background. Wild-type BALB/c mice are resistant to thyroiditis induced by immunization with mouse Tg and adjuvant (for example lipopolysaccharide). However, Treg depletion before immunization with Tg and adjuvant induced mild thyroiditis and Tg antibodies in BALB/c mice [5]. In the present study, we tested the hypothesis that Treg depleted Hi-expressor A-subunit transgenics primed with TSHR A-subunit protein plus adjuvant and boosted with A-subunit adenovirus would develop thyroiditis, thyroid damage and antibodies to Tg, as well as antibody spreading to another thyroid antigen, TPO.

## Materials and Methods

### Mouse strains

Transgenic mice expressing high and low levels of the human TSHR A-subunit in the thyroid gland (Hi-expressors and Lo-expressors) and wild-type littermates were bred at Cedars-Sinai Medical Center. Generation, characterization and breeding of these transgenics was described previously [1], [2]. The transgenics were maintained as heterozygotes by breeding them with wild-type BALB/cJ mice (Jackson Laboratories, Bar Harbor, Maine). In the present study, Lo-transgenic mice had been crossed to BALB/c for 15 or more generations and Hi expressors for 5–8 generations. The Mutant Mouse Regional Resource Center, University of California, Davis has cryopreserved the Lo- and Hi- expressor transgenics [designated C.Cg-Tg(TG-TSHR)51.9Smcl, #014125) and C.Cg_Tg(TG_TSHR)50.6Smcl/Mmucd #030109-UCD, respectively].

### Reagents for immunization

**Figure 1:**
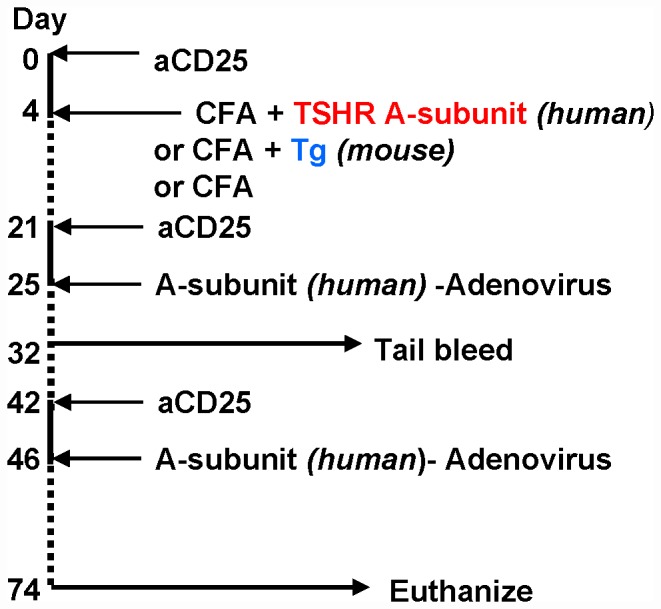
Protocol for breaking tolerance to the human TSHR in Hi-expressor A-subunit transgenics by priming with one injection of CFA+TSHR A-subunit protein CFA followed by boosting twice with A-subunit-adenovirus. Parallel studies were performed in Lo-expressor transgenics and wild-type littermates. Four days before each immunization, mice were injected with anti-CD25 to deplete regulatory T cells (Treg). The same protocol was used in a second set of mice to test the outcome of immunization with CFA+mouse Tg protein or, in a third set, with CFA alone.

a. Anti-CD25 was kindly provided by Dr. Y. Nagayama, Nagasaki University, Nagasaki. Ascites was generated from rat hybridoma (PC61, American Tissue Culture Collection) in nude mice. The antibody was purified using protein G and tested in BALB/c mice (Charles River Laboratory Inc, Yokohama, Japan) as described [6], [7]. These studies were conducted according to the Guideline for the Care and Use of Laboratory Animals, Nagasaki University. Treg depletion in Los Angeles was performed by injecting mice with 500 µg anti-CD25 intraperitoneally (ip) four days prior to immunization with protein+CFA or adenovirus immunization (Fig. 1).

b. TSHR-A-subunit protein (TSHR-289) is the recombinant A-subunit expressed in Chinese Hamster Ovary (CHO) cells, purified by affinity chromatography [8] and dialyzed against 10 mM Tris, 50 mM NaCl, pH 7.4, before use.

c. Mouse Tg (mTg) was isolated by homogenizing murine thyroid glands (Pel-Freez, AR) in phosphate buffered saline, precipitated with 45% saturated (NH_4_)SO_4_, followed by dialysis and fractionation on a Sephadex G-200 column [9].

d. Complete Freund's Adjuvant (CFA) was obtained from Sigma Aldrich (F5881; St. Louis, MO).

e. A-subunit adenovirus and control-adenovirus:- Adenovirus encoding the human TSHR A-subunit (A-sub-Ad) and adenovirus lacking an insert (“Con-Ad) were previously described [1], [10]. Both viruses were propagated in HEK293 cells, purified by CsCl density gradient centrifugation and viral particle concentration determined by absorbance at 260 nm, as described [11]. Immunization was performed by injecting 5×10^10^ particles (in 50 µl) intramuscularly (im).

### Immunization protocol

Lo- and Hi A-subunit expressor transgenics and wild-type littermates were immunized as follows (Fig. 1):-

- Day 0: anti-CD25 (500 µg) ip.
- Day 4: human TSHR-A-sub protein (50 µg) emulsified 1∶1 with CFA, injected subcutaneously (sc). A second set of transgenic- and wild-type mice was immunized according to the same protocol using CFA+Tg (mouse Tg, 40 µg), and a third set was immunized using CFA alone (emulsified with phosphate buffered saline without protein).
- Day 21: anti-CD25 (500 µg ip).
- Day 25: A-sub-Ad im.
- Day 42: anti-CD25 ip (500 µg).
- Day 46: A-sub-Ad-Ad im.
- Day 74: euthanasia.

Another group of WT mice was immunized three times with A-sub-Ad without anti-CD25 or CFA. Controls were WT mice immunized with adenovirus lacking an insert (Ad-Con) [10].

All animal studies (including breeding and immunization) were approved by the Institutional Animal Care and Use Committee at Cedars-Sinai Medical Center and performed with the highest standards of care in a pathogen-free facility.

### TSHR antibodies

Three assays were used to measure TSHR antibodies:-

- TSHR ELISA antibody binding was evaluated using ELISA wells coated with purified TSHR A-subunit protein (5 µg/ml, see above) as described [12]. Duplicate serum aliquots (1∶100 dilution) were added to the wells and binding was detected with horseradish peroxidase-conjugated mouse anti-IgG (Sigma Aldrich, St. Louis, MO). The signal was developed with o-phenylenediamine and H_2_O_2_ and the optical density (OD) read at 490 nm. The IgG subclass distribution of TSHR antibodies was determined using A-subunit (5 µg/ml)-coated ELISA wells and goat anti-mouse antibodies specific for mouse IgG1, IgG2a and IgG2b (Caltag, Laboratories, Burlingame CA). As for IgG class antibodies, the signal was developed with o-phenylenediamine and H_2_O_2_. The data for IgG class antibodies and IgG subclasses are reported as the OD at 490 nm.
- Inhibition of TSH binding to the TSHR (TSH binding inhibition, TBI) using a commercial kit (Kronus, Boise, ID). Aliquots (25 µl) of mouse serum were incubated with detergent solubilized TSHR; ^125^I-TSH was added and the TSHR-antibody complexes were precipitated with polyethylene glycol. TBI values were calculated from the formula:-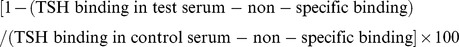

- Linear TSHR antibody epitopes: TSHR peptides: Twenty six peptides corresponding to amino acids in the *human* TSHR extracellular domain and three extracellular loop peptides [13] were kindly provided by Dr. John Morris (Mayo Clinic, Rochester MN). Ectodomain peptides are 20 amino acids long and overlap the subsequent peptide by 5 residues. These peptides are referred to as A to Z and EC1-EC3 for ectodomain and extracellular loop peptides, respectively (Table 1). In addition, peptides for the region downstream of the A-subunit up to the cell membrane in the *mouse* TSHR [14] were purchased from Peptide 2.0 (Chantilly, VA).

**Table 1:** TSHR peptides used to investigate recognition of linear antibody in the present and previous studies (for example [15]).

Peptide	Residues	TSHR amino acids	hu/mo
A	22–41	MGCSSPPCECHQEEDFRVTC	hu
B	37–56	FRVTCKDIQRIPSLPPSTQT	“
C	52–71	PSTQTLKLIETHLRTIPSHA	“
D	67–86	IPSHAFSNLPNISRIYVSID	“
E	82–102	YVSIDVTLQQLESHSFYNLS	“
F	97–116	FYNLSKVTHIEIRNTRNLTY	“
G	112–131	RNLTYIDPDALKELPLLKFL	“
H	127–146	LLKFLGIFNTGLKMFPDLTK	“
I	142–161	PDLTKVYSTDIFFILEITDN	“
J	157–176	EITDNPYMTSIPVNAFQGLC	“
K*	172–191	FQGLCNETLTLKLYNNGFTS	hum & mo
L	187–206	NGFTSVQGYAFNGTKLDAVY	hu
M	202–221	LDAVYLNKNKYLTVIDKDAF	“
N	217–236	DKDAFGGVYSGPSLLDVSQT	“
O	232–251	DVSQTSVTALPSKGLEHLKE	“
P	247–266	EHLKELIARNTWTLKKLPLS	“
Q*	262–281	KLPLSLSFLHLTRADLSYPS	hu & mo
R*	277–296	LSYPSHCCAFKNQKKIRGIL	hu & mo
	289	end A-sub (immunogen)	
S	292–311	IRGILESLMCNESSMQSLRQ	hu
		IRGILESLMCNESS**RN**SLRQ	mo
T	307–326	QSLRQRKSVNALNSPLHQEY	hu
		**RN**LRQRKSVNAL**RG**P**IY**QEY	mo
U	322–341	LHQEYEENLGDSIVGYKEKS	hu
		**IY**QEYEE**DP**GD**NS**VGYK**QN**S	mo
V	337–356	YKEKSKFQDTHNNAHYYVFF	hu
		YK**QN**SKFQ**ESPSNS**HYYVFF	mo
W	352–371	YYVFFEEQEDEIIGFGQELK	hu
		YYVFFEEQEDE**VV**GFGQELK	mo
X	367–386	GQELKNPQEETLQAFDSHYD	hu
		GQELKNPQEETLQAF**E**SHYD	mo
Y	382–401	DSHYDYTICGDSEDMVCTPK	hu
		**E**SHYDYTICGD**N**EDMVCTPK	mo
Z*	397–415	VCTPKSDEFNPCEDIMGYK	hu & mo
EC1*	474–494	DLYTHSEYYNHAIDWQTGPGC	hu & mo
EC2	561–570	SSYAKVSICLPMDTETPLAL	hu
		SSYAKVSICLPMDT**D**TPLAL	mo

Ectodomain peptides are referred to as A-Z and extracellular loop peptides as EC1-2. Peptides corresponding to the *mouse* TSHR (14) are included from the end of the A-subunit (the immunogen; residue 289 to the membrane and EC1 and EC2. Abbreviations:- hu, human TSHR residues; mo, mouse TSHR residues;

* identical peptides (human and mouse, hu & mo); amino acid differences in the mouse TSHR in bold.

As previously described [15], ELISA wells were coated with individual peptides (10 µg/ml) by incubation in 35 mM NaHCO_3_, 15 mM Na_2_CO_3_, pH 9.3. Test sera (duplicates, diluted 1∶100 or 1∶500 in high titer sera) were incubated on peptide-coated wells and reactivity developed with anti-mouse IgG and substrate. The data are presented as the OD value for binding to a particular peptide. Peptide assays included A-subunit protein coated wells and normal mouse serum as negative and positive controls, respectively.

### Autoantibodies to mouse Tg

Serum binding to mouse Tg was measured by ELISA as previously described [16]. Briefly, ELISA wells were coated with mouse Tg (1 µg/ml; as described above). Test sera were diluted 1∶100, antibody binding was detected with horseradish peroxidase-conjugated mouse anti-IgG (Sigma Aldrich) and the signal was developed with o-phenylenediamine and H_2_O_2_. The positive control was serum from a NOD.H2h4 mouse that developed mouse Tg antibodies on high iodide intake [16]; the negative control was serum from unimmunized BALB/c mice. The data are expressed as OD 490 nm.

### Autoantibodies to mouse TPO

Serum binding to mouse TPO was measured by flow cytometry as previously described [16] using Chinese hamster ovary (CHO) cells stably expressing mouse-TPO [16]. Mouse sera (1∶50 dilution) were incubated with mouse-TPO-CHO cells and binding was detected using fluorescein isothyocyanate-conjugated affinity purified goat anti-mouse IgG (Life Techologies, Grand Island, NY). Cells staining with propidium iodide (1 µg/ml) were excluded from analysis. Flow cytometry for IgG class antibody binding to mouse TPO included serum from unimmunized BALB/c mice (negative control). The positive control was mouse monoclonal antibody (#15) that recognizes mouse TPO [16]. Flow cytometry was performed (10,000 events) using a FACScan with CELLQUEST Software (Becton Dickinson, San Jose, CA). Data are reported as the geometric mean (Geo Mean).

### Serum thyroxine and thyroid histology

Total thyroxine (T4) was measured in undiluted mouse serum (25 µl) by radioimmunoassay using a kit (Siemens Healthcare Diagnostics, Los Angeles, CA). Control values (mean+2SD). Thyroids were fixed in buffered formaldehyde (pH 7.4), paraffin-embedded and serial sections were stained with hematoxylin and eosin (Research Animal Diagnostic Laboratory, University of Missouri, Columbia, MO).

### Statistical Analyses

The statistical significance of differences between the magnitude of responses in multiple groups was determined by ANOVA and testing between two groups by Mann Whitney Rank Sum test or, when normally distributed, by Student's t test.

## Results

Hi- and Lo-expressor transgenics and wild-type (WT) mice were depleted of Treg using anti-CD25 4 days before “priming” with CFA+TSHR A-subunit protein and again before each of two boosts with A-subunit-adenovirus (A-sub-Ad). In parallel, a second set of the three mouse strains was primed with CFA+Tg and a third set with CFA alone, followed by boosting with two A-subunit-Ad injections (Fig. 1). Sera were tested for antibodies to the TSHR, Tg and TPO on two occasions:- one week after the first A-sub-Ad injection (day 32) and a month after the second A-sub-Ad injection (euthanasia, day 74). Thyroid lymphocytic infiltration (thyroiditis) and serum T4 were examined at euthanasia. These time intervals reflect the outcome in CFA+A-subunit protein primed mice of a *single* or *two* boosts of A-sub-Ad (days 32 and 74, respectively).

CFA priming (with or without protein) had unanticipated effects on the outcome of immunization. Therefore, we consider first the development of antibodies to the immunogens, namely the TSHR A-subunit and in some cases Tg, before considering development of antibodies to TPO, thyroiditis and serum T4.

### Induced TSHR antibodies

**Figure 2:**
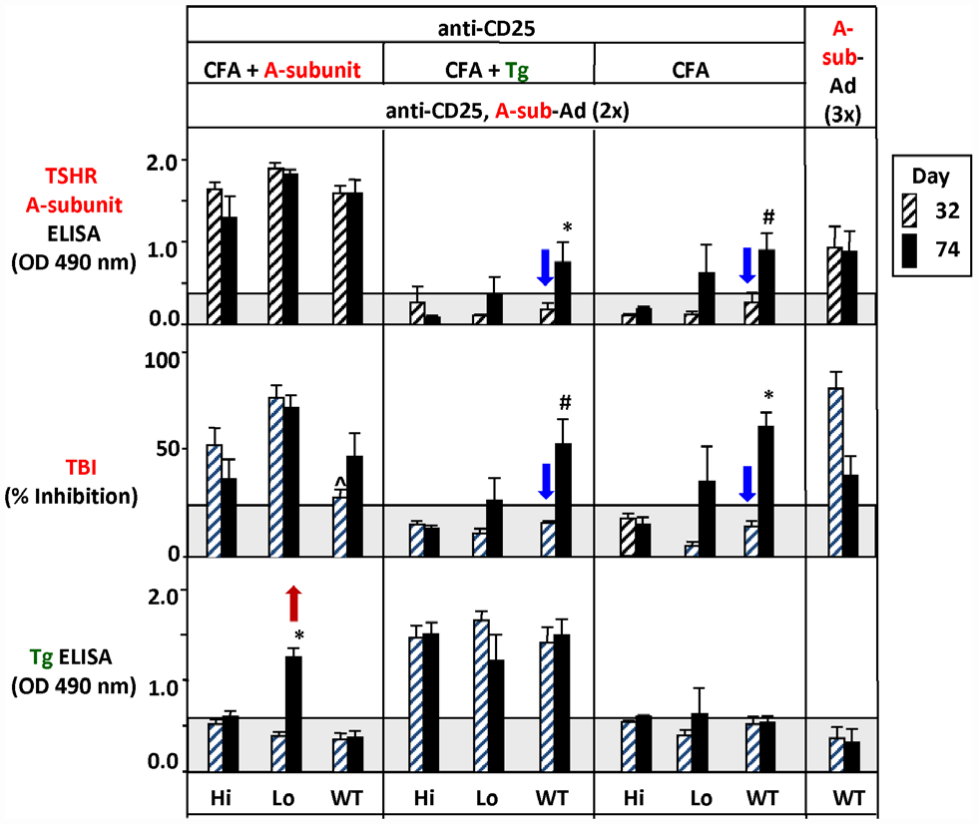
Antibodies to the TSHR and Tg induced in transgenic mice expressing high or low levels of the human TSHR A-subunit (Hi - and Lo- expressors, respectively) and wild-type littermates. Mice were depleted of CD25-positive cells before priming with (a) CFA+TSHR A-subunit protein; (b) CFA+mouse Tg or (c) CFA alone. Subsequently, mice received two cycles of anti-CD25+ depletion 4 days before immunization with A-sub-Ad. Data shown are the Mean+SEM (n = 5–7 mice) in sera drawn after 32 days (striped bars) and after 74 days (black bars; euthanasia). TSHR protein and TSHR antibodies (ELISA and TBI) are indicated in red; mouse Tg and mouse Tg antibodies are indicated in green. The grey areas indicate the mean ± 2 SEM for wild-type mice immunized with control-adenovirus (n = 4). Statistical analyses:- *Upper panel*-TSHR ELISA antibody: Significantly greater in WT than Hi-expressor mice at day 74 * p = 0.004; ^#^, p = 0.016 (rank sum test). *Middle panel* - TBI: significantly greater in WT mice after 65 days than in Hi-expressors at day 65 # p<0.05 (ANOVA), * p<0.001 (t test); ∧ significantly lower than in WT mice immunized three times with A-sub-Ad p<0.001 (t test). *Lower panel* - Tg antibody: *, p<0.05 versus Hi-expressor and WT mice (ANOVA, p<0.5).

High levels of TSHR antibody measured by ELISA developed in all three strains primed with CFA+TSHR A-subunit protein and boosted once or twice with A-sub-Ad (Table 2; Fig. 2, upper panel, left). As would be anticipated for a potent immunization protocol, namely CFA+A-subunit protein, these ELISA levels were higher than in WT mice immunized without CFA but, rather, three times with A-sub-Ad and without Treg depletion (Fig. 2, upper panel, right). Measuring TSHR antibodies by TSH binding inhibition (TBI) rather than by ELISA provided similar results in all three strains primed with CFA+A-subunit protein, but with some differences. Unlike the similar TSHR ELISA antibody levels (Table 2, Fig. 2, upper panel, left), TBI levels were highest in Lo-expressor transgenics and significantly greater than in WT littermates after a *single* A-sub-Ad immunization (Fig. 2, middle panel, left). Moreover, TBI levels for WT mice primed with CFA+A-subunit protein and boosted *once* with A-sub-Ad were significantly lower than TBI values in WT mice immunized with A-sub-Ad (three times) without Treg depletion (middle panel, extreme left versus right).

**Table 2:** Antibodies to the TSHR and Tg induced in transgenic mice expressing high or low levels of the human TSHR A-subunit (Hi - and Lo- expressors) and wild-type (WT) littermates.

	Day	Hi-Expressor	Lo-Expressor	WT	WT
		aCD25 (2×)	aCD25 (2×)	aCD25 (2×)	None
		A-sub-Ad (2×)	A-sub-Ad (2×)	A-sub-Ad (2×)	A-sub-Ad (3×)
Priming		CFA/A-sub	CFA/A-sub	CFA/A-sub	None
TSHR ELISA	32	**1.64**±0.09	**1.89**±0.07	**1.60**±0.09	**0.92**±0.26
(OD 490 nm)	74	**1.29**±0.27	**1.83**±0.06	**1.59**±0.17	**0.87**±0.25
TBI	32	**54.5**±8.5	**77.9**±5.7	**29.2**±3.8	**81.6**±8.3
(% inhibition)	74	**38.1**±9.8	**73.1**±5.8	**48.8**±11.8	**39.1**±9.4
TgAb	32	0.51±0.05	0.39±0.05	0.35±0.06	0.37±0.12
(OD 490 nm)	74	0.60±0.06	**1.25**±0.10	0.37±0.07	0.32±0.15

Priming		CFA/Tg	CFA/Tg	CFA/Tg	None*
TSHR ELISA	32	0.26±0.20	0.10±0.01	0.18±0.07	**0.92**±0.26
(OD 490 nm)	74	0.08±0.02	0.36±0.21	**0.75**±0.24	**0.87**±0.25
TBI	32	15.4±1.5	11.0±2.2	16.2±0.9	**81.6**±8.3
(% inhibition)	74	13.1±1.7	27.1±10.9	**54.5**±12.2	**39.1**±9.4
TgAb	32	**1.47**±0.13	**1.65**±0.11	**1.41**±0.18	0.37±0.12
(OD 490 nm)	74	**1.51**±0.11	**1.22**±0.28	**1.50**±0.17	0.32±0.15

Priming		CFA/PBS	CFA/PBS	CFA/PBS	None*
TSHR ELISA	32	0.11±0.02	0.12±0.04	0.26±0.12	**0.92**±0.26
(OD 490 nm)	74	0.19±0.03	**0.62**±0.36	**0.90**±0.20	**0.87**±0.25
TBI	32	21.6±0.1	5.0±2.1	14.3±2.8	**81.6**±8.3
(% inhibition)	74	21.0±0.4	**36.2**±17.5	**63.3**±6.9	**39.1**±9.4
TgAb	32	0.54±0.17	0.39±0.06	0.51±0.09	0.37±0.12
(OD 490 nm)	74	0.60±0.02	0.63±0.26	0.53±0.08	0.32±0.15

The data are shown graphically in Fig. 2. Values are the Mean+SEM (5–7 mice) in sera drawn after 32 and 74 days (euthanasia). The upper limits (mean+2 SEM) for wild-type mice immunized with control-adenovirus (n = 4): TSHR ELISA 0.35 OD; TBI, 26% inhibition; TgAb 0.6 OD. Statistically significant differences are shown in Fig. 2. Bold indicates positive values. None*: data for mice immunized 3× with A-sub-Ad are repeated to facilitate comparison with mice primed with CFA/Tg and CFA/PBS.

Unexpectedly, in all mouse strains primed with CFA *without* TSHR A-subunit protein (CFA+Tg or CFA alone), boosting *once* with A-subunit-Ad did not induce TSHR antibodies measured by ELISA or TBI. After a *second* A-sub-Ad boost, WT mice and some Lo- (but not Hi) expressor transgenics developed low levels of TSHR ELISA antibodies and TBI became detectable in WT mice (Fig. 2, upper panel, middle; Table 2). These greatly reduced TSHR antibody levels following CFA priming in the absence of A-subunit protein contrast with (a) the high levels in WT mice that did not receive CFA but were immunized three times with A-sub-Ad and without Treg depletion (Fig. 2, extreme right; Table 2); and (b) previous observations by others and ourselves of similar or enhanced TSHR antibody levels after Treg depletion [2], [6].

### Antibodies to mouse Tg and TPO, thyroiditis and serum T4

**Figure 3:**
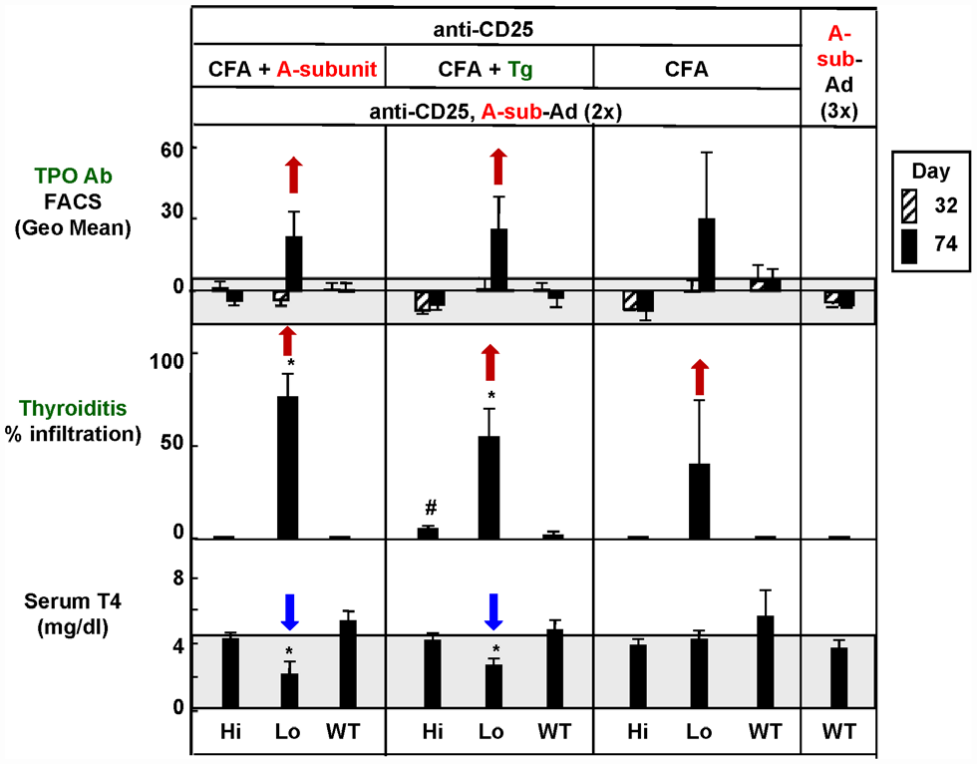
Antibodies to TPO, thyroiditis and serum T4 in Hi-expressor transgenics, Lo-expressor transgenics and WT mice immunized with CFA+TSHR A-subunit protein, CFA+mouse Tg, or CFA alone, followed by two immunizations with A-sub-Ad (see legend to **Fig. 2**). Four days prior to each of the three immunizations, mice were treated with anti-CD25 to deplete regulatory T cells. Data shown are the Mean+SEM (n = 5–7 mice) in sera drawn after 32 days (striped bars) and 74 days (euthanasia; black bars). The grey areas indicate the mean ± 2 SEM for wild-type mice immunized with control-adenovirus (n = 4). Statistical analysis:- *Middle panel*- Thyroiditis: * significantly greater in Lo expressors primed with CFA+A-sub, Tg or PBS than in Hi-expressor transgenics or wild-type mice (ANOVA on ranks, p<0.05); #, significantly greater in Hi-expressors primed with CFA+Tg than in the same strain primed with CFA+A-sub or CFA+PBS (ANOVA on ranks, p<0.05). *Lower panel*: * Serum T4 is significantly lower in Lo-expressor transgenics immunized with CFA+A-sub or CFA+Tg than in Hi-expressor transgenics or wild-type mice (p<0 05, ANOVA).

Antibodies to Tg were induced and were detectable at high levels in all three mouse strains primed with mouse CFA+Tg and subsequently immunized with A-sub-Ad (Fig. 2, lower panel; Table 2). At euthanasia (but not at day 32), antibodies to Tg were detected in Lo-expressor transgenics primed with CFA+A-subunit even though the mice had not been immunized with Tg. (Fig. 2, lower panel, left; red arrow). Similarly, TPO antibodies, absent at day 32 (Table 3) were present at euthanasia in some Lo-expressor transgenics primed with CFA+A-subunit protein, CFA+Tg or CFA alone (Fig. 3, upper panel, respectively). The presence of TPO antibodies correlated with spontaneous development of Tg antibodies in Lo-expressors primed with CFA+A-subunit protein (Table 2, Fig. 2, lower left panel). In addition, Lo-expressor transgenics primed with CFA+A-subunit or CFA+Tg had significantly lower serum T4 levels at euthanasia than wild-type or Hi-expressor mice immunized in the same way (Fig. 3, bottom panel; Table 3). The decreased T4 levels in Lo-expressor transgenics reflect thyroid damage leading to antibody spreading to other thyroid antigens, namely Tg (in mice not primed with Tg) and TPO.

**Table 3:** TPO antibodies, thyroiditis and serum T4 in Hi-expressor transgenics, Lo-expressor transgenics and WT mice.

	Day	Hi-Expressor	Lo-Expressor	WT	WT
		aCD25 (2×)	aCD25 (2×)	aCD25 (2×)	
		A-sub-Ad (2×)	A-sub-Ad (2×)	A-sub-Ad (2×)	A-sub-Ad (3×)
Priming		CFA/A-sub	CFA/A-sub	CFA/A-sub	None
TPO Ab	32	1.1±2.9	−3.8±2.6	0.4±3.0	−4.6±0.8
(Geo Mean)	74	−4.0±2.2	**22.4**±11.0	−0.3±3.6	−5.9±0.7
Thyroiditis (%)	74	0.1±0.0	**76.4**±12.9	0.1±0.0	0.1±0.0
T4 (µg/dL)	74	4.1±0.4	*2.0*±0.8	5.1±0.6	3.9±1.0

Priming		CFA/Tg	CFA/Tg	CFA/Tg	None*
TPO Ab	32	−7.9±1.4	1.5±6.0	1.8±3.2	−4.6±0.8
(Geo Mean)	74	−5.5±2.2	**24.6**±19.6	−3.4±4.9	−5.9±0.7
Thyroiditis (%)	74	4.4±1.3	**50.0**±16.7	2.1±2.0	0.1±0.0
T4 (µg/dL)	74	4.0±0.4	*2.5*±0.4	4.6±0.6	3.9±1.0

Priming		CFA/PBS	CFA/PBS	CFA/PBS	None*
TPO Ab	32	−7.7±0.6	−0.4±4.8	4.3±6.5	−4.6±0.8
(Geo Mean)	74	−8.4±4.1	**29.9**±28.0	4.1±5.0	−5.9±0.7
Thyroiditis (%)	74	0.4±0.3	**52.5**±19.0	0.1±0.0	0.1±0.0
T4 (µg/dL)	74	3.7±0.4	4.1±0.5	5.4±1.6	3.9±1.0

The data are shown graphically in Fig. 3. Values are the Mean ± SEM (5–7 mice) in sera drawn after 32 and 74 days (euthanasia). The upper limit (mean+2 SEM) for wild-type mice immunized with control-adenovirus (n = 4) are:- TPO Ab 15 Geo mean; Thyroiditis, 0.1%; T4, 4.3 µg/dL. Statistically significant differences are shown in Fig. 3. Bold indicates positive values; italics indicate decreased serum T4. None*: data for mice immunized 3× with A-sub-Ad are repeated to facilitate comparison with mice primed with CFA/Tg and CFA/PBS.

A low level of thyroid lymphocyte infiltration occurred in Hi-expressor transgenics primed with CFA+Tg (Fig. 3), but not in the same strain primed with CFA+A-subunit or CFA alone. However, there was no evidence in these transgenics for the development of antibodies to Tg or TPO and T4 levels were similar to those in WT littermates immunized in the same way (Fig. 2 and 3; Tables 2 and 3).

### TSHR antibody subclasses

**Figure 4:**
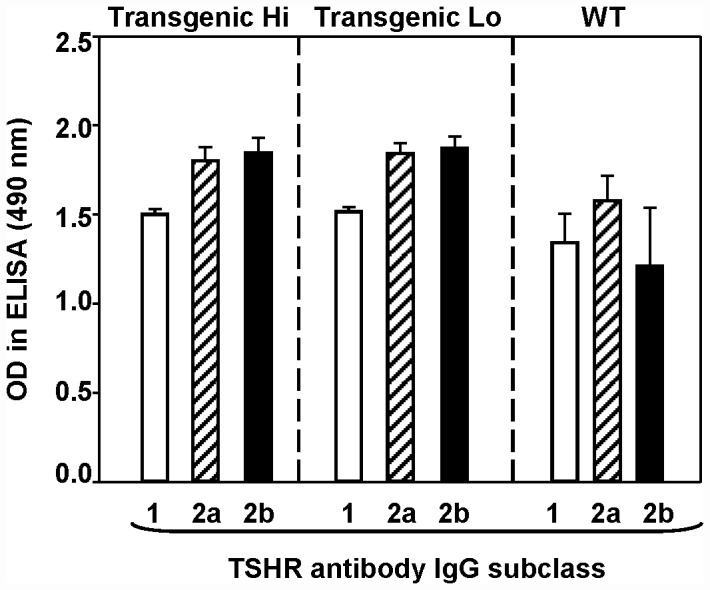
IgG subclasses of TSHR antibodies in Hi- and Lo expressor A-subunit transgenic mice and WT littermates injected with anti-CD25 before immunization with TSHR A-sub protein+CFA and two subsequent injections of human TSHR A-subunit-adenovirus. ELISA plates were coated with TSHR A-subunit protein and serum binding was detected using goat anti-mouse IgG1, IgG2a and IgG2b. Data are shown for the mean+SEM OD 490 nm in ELISA for 4 WT mice, 7 Lo-expressor transgenics and 5 Hi-expressor transgenics.

The IgG subclass distribution of TSHR antibodies was determined to investigate differences between transgenic mice that could possibly contribute to the lack of thyroiditis in Hi expressor mice primed with CFA+A-subunit protein. However, there were no significant differences in the levels of A-subunit antibodies of IgG1 (reflecting T helper 2 cytokines) or IgG2a and IgG2b (reflecting T helper 1 type cytokines) in Hi- and Lo-expressor mice or in WT littermates (Fig. 4).

### Linear TSHR antibody epitopes

**Figure 5:**
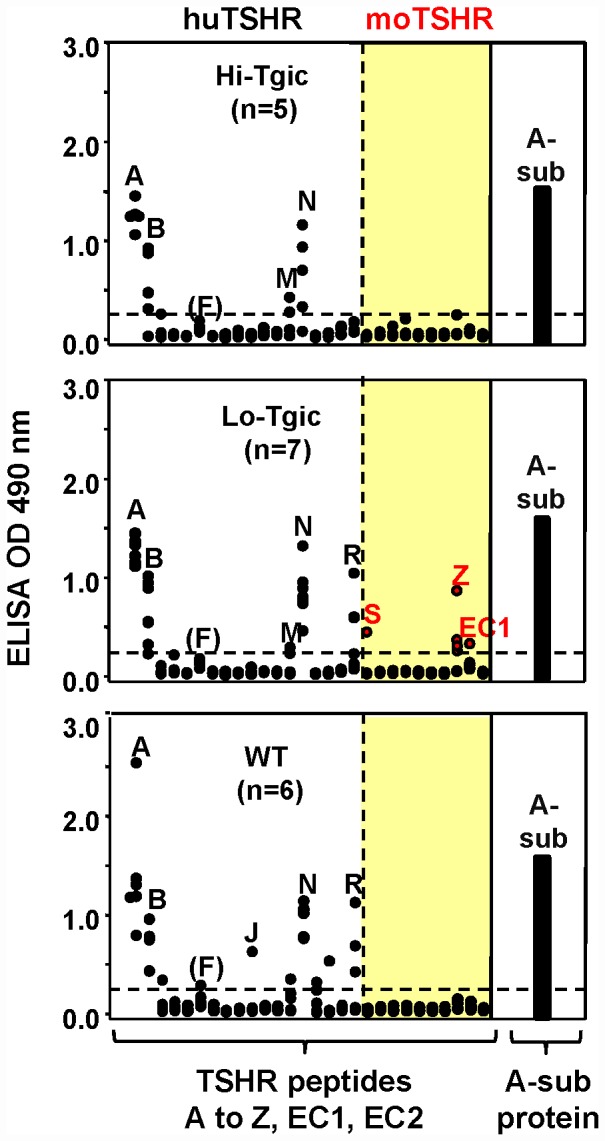
Linear TSHR antibody epitopes recognized by Hi- and Lo expressor A-subunit transgenic mice and wild-type littermates injected with anti-CD25 before immunization with TSHR A-sub protein+CFA and two subsequent injections of human TSHR A-subunit-adenovirus. Antibody binding was studied to ELISA wells coated with synthetic peptides encompassing the human TSHR ectodomain (peptides A to Z) and two extracellular loops (EC1 and EC2)(amino acid sequences in Table 1). A dashed line indicates the last peptide (“R”) in the human TSHR A-subunit. The shaded area (to the right) includes the *human* peptides downstream of the A-subunit (S to Z, EC1 and E2); antibody binding to peptides in this region implies recognition of the *mouse* TSHR. The data for individual mice are shown as the OD binding value (490 nm) for each peptide (black circles) and to TSHR A-sub protein (black bar). The number of mice studied in each group is given in parentheses.

TSHR antibodies detectable by ELISA in mice primed with CFA+TSHR A-subunit protein and boosted with A-sub-Ad were tested for recognition of a panel of peptides corresponding to the *human* TSHR ectodomain sequence (Table 1). Sera from wild-type mice as well as Lo- and Hi-expressor transgenics bound strongly to N-terminal peptides A and B as well as to several other peptides within the human A-subunit (Fig. 5). Importantly, unlike wild-type and Hi-expressor transgenics, antibodies in Lo-expressor transgenics also bound to ectodomain peptides *downstream* of the A-subunit, namely peptides S, Z and EC1. It should be emphasized that the mice were primed with *human* A-subunit protein and boosted with *human* A-sub-Ad. Consequently, binding to peptides S, Z and EC1 implies recognition of the *mouse* TSHR. Because some of these peptides differ in mice and humans (Table 1), synthetic peptides corresponding to the non-identical *mouse* peptides were purchased. Comparison of binding to the mouse peptides was similar to binding to human peptides and no additional linear epitopes were identified (data not shown).

## Discussion

Previously we found that massive thyroiditis, associated with thyroid damage and thyroid antibody spreading to Tg and TPO, was induced by Treg depletion using anti-CD25 before immunization with TSHR adenovirus in transgenic mice expressing low, but not high, levels of the human TSHR A-subunit in the thymus and thyroid [2], [4]. The goal of the present study was to determine if a potent immunization protocol, namely complete Freund's Adjuvant (CFA)+A-subunit protein, followed by boosting with A-sub-adenovirus in Treg depleted animals could break tolerance and induce thyroiditis in Hi-expressor transgenics. Although anti-CD25 depletion could also deplete activated T cells (for example [17]), all mice received the same treatment and, with some exceptions attributed to CFA rather than to anti-CD25 (described below), developed strong antibody responses.

In Treg depleted Hi-expressor transgenics, priming with CFA+A-subunit protein prior to A-subunit adenovirus immunization effectively induced TSHR antibodies at much higher levels than we observed even using high-dose TSHR-adenovirus [1], [3]. Indeed, the antibody levels were comparable with those previously achieved when these mice were primed with CFA+ A-subunit protein and boosted twice with incomplete Freund's adjuvant+A-subunit protein [1]. Despite the induction of strong antibody responses, neither the Hi-expressor transgenics nor the WT littermates developed thyroiditis. In contrast, thyroiditis developed in Lo-expressor transgenics in association with autoantibodies to Tg in all mice and autoantibodies to TPO in some animals.

The time-line for the development of antibodies to the TSHR, Tg and TPO is of potential interest. However, it should be emphasized that TSHR antibodies develop spontaneously in humans, but not in mice. In the current investigation, as in other studies (reviewed in [18]), TSHR antibodies are detected after two immunizations with the TSHR (protein or expressed by *in vivo* by adenovirus). Antibodies to Tg and TPO were only observed at euthanasia, not at the earlier time point, in Lo-expressor transgenics that developed thyroiditis. In NODH.2h4 mice, Tg autoantibodies appeared first followed some time later by TPO autoantibodies [16]. Taken together, these observations suggest that thyroid lymphocytic infiltration and thyroid damage, at least as induced by the protocol involving priming with CFA and A-subunit protein or Tg, is followed initially by autoantibodies to Tg and at a later time point by antibodies to TPO.

The basis for the lack of thyroiditis in WT mice and Hi-expressor transgenics (both on the BALB/c background) is only partially understood. Unlike Lo-expressor transgenics, WT mice do not express peptides from the *human* TSHR in the thyroid that can be recognized by T cells activated by *human* A-subunit-Ad [4]. However, BALB/c mice develop mild thyroiditis after Treg depletion and immunization with adjuvant (lipopolysaccharide)+murine Tg [5]. Therefore, we were surprised to find that priming with CFA+ murine Tg followed by A-sub-Ad did not lead to thyroiditis in WT mice.

Hi-expressor transgenics should express large amounts of human TSHR peptides in the thyroid gland. Moreover the protocol that we used effectively broke self tolerance to the human A-subunit, at least in terms of TSHR antibodies. Immunization with CFA and adenovirus would be expected to induce cytokines of T helper 1 but not T helper 2 responses, interferon-gamma and interleukin-4, respectively [19], [20]. Cytokine responses were not measured but TSHR antibodies of subclasses IgG2a and IgG2b were produced in all three mouse strains, consistent with production of the Th1 cytokine IFN-gamma. Although there may be other differences in the immune response of the Hi-expressors, it is possible that unknown thyroid characteristics may contribute to the inability to induce thyroiditis in these animals.

Why were T4 levels not elevated in WT mice that developed TBI activity after priming with CFA+A-subunit protein? As we reported previously, pre-treatment with A-subunit protein (without CFA) diverted the development of thyroid stimulating antibodies to non-functional antibodies and attenuated hyperthyroidism [21]. In addition, we used a high dose of A-sub-Ad to break tolerance in Hi-expressors, which we previously observed also diverted TSHR antibodies towards the non-stimulatory variety [22]. However, among the WT mice that received CFA only (without A-subunit protein), hyperthyroidism developed in a single mouse (T4 11.1 µg/dL), as reflected in the higher (on average) T4 levels and the wide SEM bar (Fig. 3).

Returning to TSHR antibodies, in previous studies immunizing WT BALB/c mice with adenovirus expressing either the full-length (*holo*) TSHR to the TSHR A-subunit, antibody binding was only observed to the peptide A, corresponding to the N-terminal amino acids of the TSHR (residues 22–41; residues 1–21 being the signal peptide) [15]. In contrast, immunization with CFA+A-subunit protein followed by IFA+A-subunit protein induced strong binding to a wider spectrum of peptides, namely peptides B, M, N and O [15]. Also in previous studies, Treg depleted WT BALB/c mice immunized with *holo*TSHR-Ad generated antibodies that recognized peptides downstream of the A-subunit (peptides W, X and Y) [4], consistent with the extended length of the immunogen. Lo-expressor transgenics subjected to the same protocol only developed antibodies to peptide X [4]. In the present study, immunization restricted to the A-subunit (whether protein+CFA or A-subunit adenovirus) induced a similar peptide recognition profile in WT mice and both transgenic lines, namely peptides A, B and N and (in some mice) peptide R.

The most interesting and novel finding in the current investigation came from Lo-expressor TSHR-A-subunit transgenics that developed thyroiditis after immunization with CFA+A-subunit protein followed by A-sub-Ad: when examined at euthanasia, sera from some of these mice had antibody binding to TSHR peptides not present in the immunogen, namely peptides S, Z and EC1 downstream of the A-subunit, and only represented by the naturally occurring *mouse* TSHR *in vivo*. These observations clearly indicate a breakdown in tolerance to the *mouse* TSHR. Moreover, the peptides recognized, albeit at low level, differ from those induced by immunization with the *holo*TSHR-Ad (see above). Sera from all mice tested for reactivity to TSHR peptides had antibodies to mouse Tg; however, there is no homology between the downstream peptides (S,Z or EC1) and mouse Tg. More importantly, the present data are the first to demonstrate antibody recognition of linear epitopes on the *mouse* TSHR.

A number of other potentially important observations were made which have implications for the role of infections in breaking self-tolerance. In particular, priming with CFA+Tg or PBS *inhibited* induction by A-subunit adenovirus of TSHR antibody measured by ELISA or TBI. This inhibition was most potent in mice with the greatest self-tolerance to the A-subunit (Hi-expressors) and was lower in WT mice. The effect could not be explained in terms of antigenic competition because it was observed in mice primed with CFA+Tg as well as in mice primed with CFA alone. On the other hand, TSHR antibodies were induced in Hi-expressor transgenics at levels far higher than previous achieved using A-subunit adenovirus without CFA+ A-subunit protein priming [1], [2].

To summarize, in transgenic mice expressing low intrathyroidal and intrathymic levels of the human TSHR A-subunit (Lo-expressors), the combination of Treg depletion plus immunization with a bacterial adjuvant+TSHR A-subunit protein followed by A-subunit adenovirus immunization broke self tolerance to the TSHR leading to thyroid lymphocytic infiltration. However, Hi- expressor trangenics, like WT BALB/c mice, remained resistant to the induction of thyroiditis. A novel finding for Lo-expressor transgenics was the spontaneous development of antibodies to the *mouse* TSHR *downstream* of the A-subunit. Unexpectedly, we found that the effect of bacterial products on the immune system is a “double-edged sword”. One the one hand, self tolerance was effectively broken as reflected in the high levels of TSHR antibodies induced by priming with A-subunit+Complete Freund's adjuvant (CFA) priming and A-subunit adenovirus immunization in Hi-expressor transgenics. On the other hand, prior immunization with CFA, an emulsion of mycobacteria in oil, strongly inhibited responses to subsequent immunization with A-subunit adenovirus. Consequently, adjuvant activity arising naturally *in vivo* after bacterial infections could break self tolerance if combined with the autoantigen. However, in the absence of the autoantigen, prior adjuvant activity may inhibit responses to autoantigens (such as the TSHR) that exhibit strong self-tolerance.
